# Regular measurements of EMF in a representative Norwegian city—constant exposure over time despite introduction of new technologies

**DOI:** 10.1007/s10661-022-10231-4

**Published:** 2022-08-19

**Authors:** Atle C. Markussen, Tone-Mette Sjoemoen, Edith Helene Unander, Lars Klaeboe

**Affiliations:** 1 Norwegian Communications Authority, Lillesand, Norway; 2 The Norwegian Radiation and Nuclear Safety Authority, Oslo, Norway

**Keywords:** Long-term measurements, ICNIRP, Exposure, Radio frequency fields, Data traffic, Base stations

## Abstract

The rapid growth of the wireless communication industry has resulted in the installation of numerous of base stations, everywhere in our surroundings. The population is exposed to Radio Frequency Electromagnetic Fields of varying frequency and strength. This, and introduction of new systems have risen public concerns regarding potential health effects from this RF-EMF exposure. The purpose of this project is to get an overview of any changes in exposure when new technologies are introduced. From June 2013 to October 2019, measurements were made at 16 measurement points in Kristiansand and surrounding areas in the same order, on the same day of the week and at the same time of day. The measurements are performed on the frequency bands 390, 450, 800, 900, 1800, 2100, 2400, and 2600 MHz. When we summed up the exposure for all the frequency bands relative to the limit values in a measuring point, the total values per measuring point showed that the exposure outdoors in most cases is less than 1‰ of the limit value. In 2017, a temporary increase was registered for most measurement points, but during 2018 the levels returned to the levels registered before 2017. During the increase, the levels were still low, around 3‰ of the limit values. The increase may be due to the fact that two mobile operators during this period made a comprehensive reconfiguration of their networks. The measurements presented in this report show that the exposure of the population is low, thousandths of the limit values, and relatively constant over time even though new technologies are introduced.

## Introduction

The rapid growth of the wireless communication industry has resulted in the installation of numerous of base stations, everywhere in our surroundings. The population is exposed to radio frequency electromagnetic fields of varying frequency and strength. This, and introduction of new systems have risen public concerns regarding potential health effects from this RF-EMF exposure. Several studies have addressed this concern as an attempt to inform the public about actual levels in the surroundings (Calvente et al., 2015; Fernández-García & Gil, 2017; Jalilian et al., 2019; Koppel & Hardell, 2022).

Development of wireless technology takes place continuously. To monitor the levels of RF-EMF over time in our surroundings, The Norwegian Communications Authority, Nkom, perform regular measurements in the city of Kristiansand, which we consider to be representative according to RF-EMF. The goal of these regular measurements is to map exposure over a period of several years. The results presented in this report includes radio frequency fields at street level in different parts of the city of Kristiansand. We assume that Kristiansand is representative of the technological development in telecommunications in all major cities in Norway. When choosing measuring points, great emphasis is placed on being able to carry out repeatable and comparable measurements over a long period of time. It is not a goal to find measuring points that have the highest possible levels, but rather places that are representative of areas where people have their everyday life.

An overall goal for the Norwegian authorities is to have an overview of all aspects of exposure to radio frequency fields, including development over time. Trends in the exposure situation is best followed by making comparable measurements over time. Using this approach, we will be able to see the consequences when new systems are introduced, old systems are phased out or used to a lesser extent.

The objective of this report is to present trends of exposure to radiofrequency electromagnetic fields from six years of regular measurements in a representative Norwegian city.

## Methods

### Measurements

The data presented include radio frequency fields at street level in different parts of Kristiansand, which we consider to be representative of the technological developments in telecommunications in all cities in Norway. When selecting measurement points, great importance is attached to being able to carry out comparable measurements over a long period of time. The measurements represent trends in the areas where people travel, not the highest possible levels. Thus, we do not have focus on the location of the base stations; it is the general exposure in the environment which is of interest. The measurements give a picture of how exposure changes over time.

### Measurement methods

The measurement method used by Nkom is based on recommendations for measuring radio frequency fields in the frequency range 9 kHz–300 GHz given by the European Committee for Electronic Communications, ECC (ECC, 2002), which is part of the Committee for European Postal and Telecommunications Administrations (CEPT). The measured value in a frequency band represents an instantaneous value for power density (expressed in watts per square meter, W/m^2^) at the time of measurement. However, it is the average value averaged over any 6-min period that should not exceed the applicable limit values, also called reference levels, for exposure from transmitters (ICNIRP, 1998). For practical reasons, the measurements are made in this way, as it is the development over time, trend, that is interesting in this project. In 2020, ICNIRP (2020) updated its guidelines. These changes were made after the measurements in this paper were completed. The measurements were performed on weekdays between 9 am and 3 pm and the measurement probe was 200 cm above the ground.

### Measuring equipment

The measurements were performed with a measuring antenna and spectrum analyzer from Rohde & Schwarz. The measuring antenna is of the TS-EMF type and covers the frequency range 30 MHz–3 GHz. The frequency bands included in the measurements are shown in Table 1.

**Table 1:** Frequency bands, reference levels, and services included in the measurements

**Frequency band, MHz**	**Reference levels (W/m**^**2**^**), ICNIRP**	**Service**
390	2	Emergency service network
450	2.25	Mobile telephony
800	4	Mobile telephony
900	4.5	Mobile telephony
1800	9	Mobile telephony
2100	10	Mobile telephony
2400	10	Wireless network/bluetooth/license-free
2600	10	Mobile telephony

### Measurement locations

Kristiansand is a medium-sized city by Norwegian standards, located in southern Norway. With suburbs, the city consists of 65,000 inhabitants and is the sixth largest city in Norway. Approximately 6000 people live in the city center. The exposure situation in Kristiansand is considered to be representative for all cities in Norway.

In order to find measuring points that could be reused over several years, different points were tested during May 2013.

**Fig. 1:**
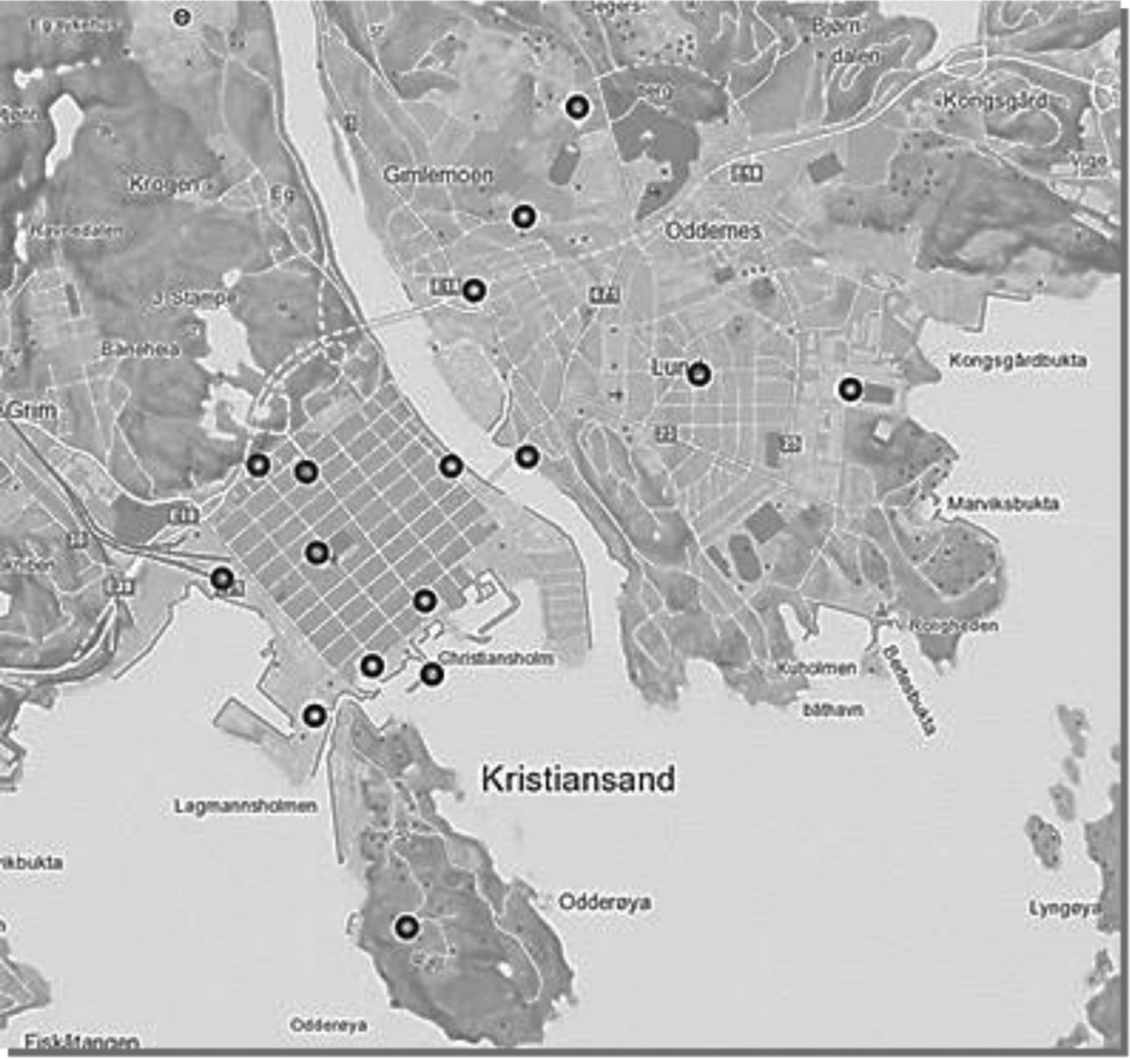
The measurement points in Kristiansand and surroundings

The following aspects were relevant in the selection:

- Measurements should be made where people usually stay:
  parks/green spaces/playgrounds, schools, parking spaces and residential areas. Figure 1 shows distribution of the measurement points in Kristiansand.
- Sufficient space for the measuring antenna and car, with the possibility of being parked for as long as necessary.
- Separate from traffic and possible parking for other cars.

From June 2013 to October 2019, measurements were made at 16 measurement points in Kristiansand and surrounding areas (Fig. 1) in the same order, on the same day of the week and at the same time of day. The first years the measurements were performed each month, later the measurements were performed every quarter. Three measurements points, 4, 10, and 13, had to be moved during the period because they of different reasons became unavailable for measurements. These three points are not included further in the analyses as the trends remained similar.

## Results

The results in this report include measurements from June 2013 to October 2019. The measurement results are stated in ‰ (thousandth) of the limit values for the population. The limit values are frequency dependent, and for power density they varies between 2 and 10 W/m^2^ for the actual frequencies. The contribution from each frequency band is specified in relation to the limit value for this frequency, and then all contributions can be summed and give a total value for exposure in ‰ of the limit values.

**Fig. 2:**
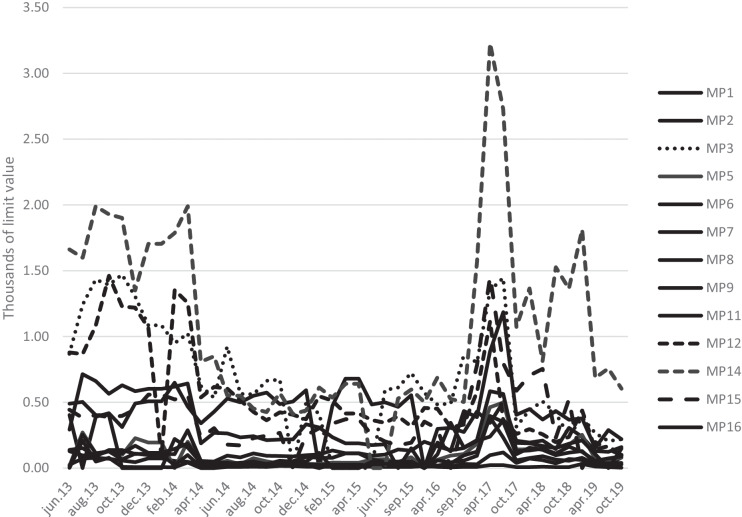
Total values of all measured frequency bands relative to the limit values for power density on different dates for each measuring point (MP). The total values were reduced at most measuring points after the implementation of LTE (4G) in the 800-MHz band in February 2014. The values close to zero are all presented as solid lines. The purpose of the figure is to show trends and how the values can vary in a city. The peak during 2017 is explained in the text

Figure 2 shows the total values of all measured frequency bands relative to the limit values for the different measurement points. The total values from all frequencies per measurement point show outdoor values up to 3 ‰ of the limit values for the general population. Zero is used here for levels below the detection limit of the instrument. This does not mean that there is no exposure at the measuring point, but it is undetectable. Most of the measurements shows values below 1‰ of the limit values. The purpose of the figure is to show the levels of exposure compared with the limit values and how values can vary in different locations in a city. The total values were reduced at most measuring points after the implementation of LTE (4G) in the 800-MHz band in February 2014.

**Fig. 3:**
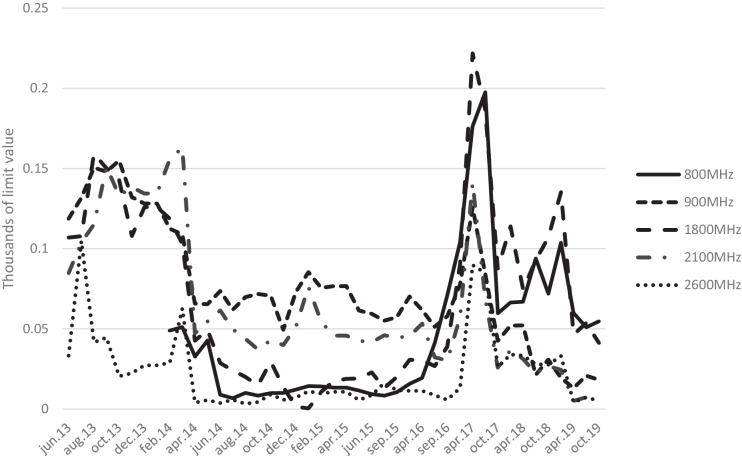
Average values for all measuring points for the various frequency bands allocated to mobile telephony relative to the limit values for each date. The measurements indicate a downward trend for all mobile systems after the implantation of LTE (4G) in the 800-MHz band was introduced in February 2014. The peak during 2017 is explained in the text

Figure 3 shows the average power density for the different frequencies for all measurement points, relative to the limit values. The measurements indicate a downward trend for all other mobile systems after the implantation of LTE (4G) in the 800 MHz band was introduced in February 2014. This trend seems to be permanent, as we cannot see any notable increase in the average power density at the end of our measuring period. The peak during 2017 is touched on in the discussion.

## Discussion

The aim of these measurements is to study the development of exposure over time. After 6 years of regular measurements, total and average values are relatively constant, despite the introduction of LTE (4G) in the 800-MHz band in 2014. The measurements described in this report show that the total values are generally below 1‰ of the limit values in places that we consider representative for outdoor exposure. Even if the measurements are not averaged over 6 min as it was recommended before 2020, we consider that the values are still representative for the trends.

This study focuses on values relative to the limit values, which we consider to be relevant because health effects are the basis of the concern. The use of power density (W/m^2^) or electric field strength (expressed in volt/meter, V/m) is not informative in this context as the limit values are frequency dependent. If 900 MHz is dominant in an area, the limit value in this area will be 4.5 W/m^2^. In areas where 2100 MHz is dominant, the limit value will be 10 W/m^2^. Both areas can have similar values in W/m^2^ or V/m, and these units provide limited information from a health perspective.

As seen from the figure, the values vas higher for some measuring points between June 13 and June 14. LTE 800 was introduced at the end of this period. With better coverage, LTE 800 took over the traffic, e.g., from UMTS (3G). The coverage is different for the different technologies, at different measuring points, so in some places it makes a bigger impact than others which results in the higher values.

The levels increased in 2017, and then fell again towards the end of the year and beyond 2018. The reason for this seems to be that two mobile operators in this period made an extensive reconfiguring of their networks. New base stations were set up at the same time as the output power at some existing base stations increased. Throughout 2017, the effect was adjusted down again. In addition, a number of UMTS base stations in Kristiansand were removed during the same period. These events coincide well with the reduced values.

Our measurements show that the values in the environment are very low compared to current limit values. The low exposure agrees well with studies in other countries (Calvente et al., 2015; Fernàdez-Garcìa & Gil, 2017; Jalilian et al., 2019; Koppel & Hardell, 2022).

Possible errors on the instruments were also evaluated as a possible reason for the increased values. The raw data for the curves showed that the increase did not occur at all measuring points during a day and that the increase was distributed over different frequency bands indicates no measure errors.

### Factors that may have influenced the measurements

Radio frequency fields vary with time and place. It varies over time, both due to variations in the environment and varying traffic load on the wireless systems. Therefore, it is important that the measurements at the individual measuring point is performed at approximately the same time of day each time. Some points are not measured every time because of parked cars and construction work. This is probably of little importance for the interpretation of the development when the measurements have been performed for many years.

The uncertainty of the measuring instrument is stated to be ± 1.5 dB. In addition, the measurements may be affected by changes in circumstances. This means that a measurement setup will not provide 100% (percent) repeatable measurement values. Furthermore, the transmitting power from some base stations varies due to the fact that the traffic load is not constant. Whether there is or not is clear view for the antennas is an important factor in how much the environment affects the measurements. In cases where there is no clear view, the level may vary greatly over time because movable elements in the environment, such as cars, can affect the reflection of the signals. With completely free visibility to the antennas, changes in the environment will not affect the measurements to the same extent. New buildings near the measuring points can affect the results, but this also reflects the variations on a certain point in a city.

## The measurements will continue

The Norwegian authorities consider it important to have an overview of the exposure of the population over time. The measurements will continue, and we have not planned any completion for this project. 5G will be included in the project when this technology is introduced in Kristiansand, and we will monitor how the total exposure is affected after the introduction.

## Conclusion

The measurements presented in this report show that the exposure of the population is low, thousands of the limit values, and relatively constant over time even though new technologies are introduced.

## Data Availability

All data generated or analyzed during this study are included in this published article.

## References

[CR1] Calvente I, Fernández MF, Pérez-Lobato R, Dávila-Arias C, Ocón O, Ramos R, Ríos-Arrabal S, Villalba-Moreno J, Olea N, Núñez MI (2015). Outdoor characterization of radio frequency electromagnetic fields in a Spanish birth cohort. Environmental Research.

[CR2] ECC. (2002). Measuring non-ionizing electromagnetic radiation (9 kHz–300 GHz). ECC Recommendation (02)04 (revised Bratislava 2003, Helsinki 2007). Copenhagen: Electronic Communication Committee (ECC). http://www.erodocdb.dk/doks/implement_doc_adm.aspx?docid=1908

[CR3] Fernández-García R, Gil I (2017). Measurement of the environmental broadband electromagnetic waves in a mid-size European city. Environmental Research.

[CR4] ICNIRP. (1998). Guidelines for limiting exposure to time-varying electric, magnetic, and electromagnetic fields (up to 300 GHz). *Health Physics*, *74*(4), 494–522. 9525427

[CR5] ICNIRP. (2020). Guidelines for limiting exposure to electromagnetic fields (100 kHz to 300 GHz). *Health Physics*, *118*(5), 483–524. 10.1097/HP.0000000000001210 32167495

[CR6] Jalilian, H., Eeftens, M., Ziaei, M., Röösli, M. (2019). Public exposure to radiofrequency electromagnetic fields in everyday microenvironments: an updated systematic review for Europe. *Environmental Research*, 176. 10.1016/j.envres.2019.05.048 31202043

[CR7] Koppel T, Hardell L (2022). Measurements of radiofrequency electromagnetic fields, including 5G, in the city of Columbia, SC, USA. World Academy of Sciences Journal.

